# NEW CLASSIFICATION FOR ESOPHAGEAL MOTILITY DISORDERS (CHICAGO CLASSIFICATION VERSION 4.0^©^) AND CHAGAS DISEASE ESOPHAGOPATHY (ACHALASIA)

**DOI:** 10.1590/0102-672020210002e1624

**Published:** 2022-01-31

**Authors:** Fernando Augusto Mardiros HERBELLA, Osvaldo MALAFAIA, Marco G. PATTI

**Affiliations:** 1 Departamento de Cirurgia, Universidade Federal de São Paulo, São Paulo, Brasil; 2 Faculdade Evangélica Mackenzie do Paraná/Instituto de Pesquisas Médicas, Curitiba, PR, Brasil; 3 Hospitais da Universidade da Carolina do Norte, Chapel Hill, NC, USA

High-resolution manometry (HRM) is undoubtedly an evolution of conventional manometry. This technology was developed at the beginning of the century, even though it reached Latin America only in 2008^2^. Esophageal motility testing became at least more comfortable and intuitive for the nonexpert after HRM; however, an abundance of new parameters and diagnosis was agreeable to the eye presentation of the colorful plots. A consensus became mandatory and a panel of experts started to release periodic guidelines for HRM interpretation, the so-called Chicago Classification. A new version has just been published^11^ with some practical implications for surgeons^3^. In this new version, the diagnosis of achalasia is still defined by abnormal relaxation of the lower esophageal sphincter (LES) as measured by an elevated integrated relaxation pressure (IRP) and the division of subtypes based on esophageal pressurization is kept unaltered. Different from the previous versions, however, some situations define an “inconclusive diagnosis of achalasia” as (a) absent contractility with no appreciable peristalsis in the setting of IRP values at the upper limit of normal; (b) evidence of appreciable peristalsis with changing position in the setting of a type I or II achalasia pattern; and (c) an abnormal IRP with evidence of spasm and evidence of peristalsis in the setting of a type III achalasia pattern. Let us discuss the implications of these assertions in the management of patients with Chagas disease esophagopathy (CDE; achalasia) since HRM is currently more disseminated in Brazil since national systems were developed^5^.

The first point for discussion is that the conclusive diagnosis of achalasia is based on aperistalsis. The all or nothing at all concept is still valid for the conclusive manometric diagnosis of achalasia. Some authors have not been applying this criterion in patients with CDE^1^. An “undetermined” phase of CDE is usually quoted as a common finding in patients with CDE^6^. Whether these cases truly represent a predisease to progress to complete aperistalsis is elusive. On the one hand, researchers who have the chance to study patients with positive serological tests for CDE before esophageal symptoms may manifest what does not occur in idiopathic achalasia. On the other hand, patients with Chagas disease may never develop CDE but may present with other esophageal diseases such as gastroesophageal reflux disease (GERD)^7^. Chicago Classification 4.0 clarified that primary esophageal motility disorders should only be considered in the absence of GERD and, as such, all these cases of an “undetermined” phase must undergo pH monitoring. One must be aware that pseudoreflux may occur in achalasia due to food fermentation in the esophagus, but tracings are very characteristic of this occurrence^8^.

The second point for discussion is that “inconclusive diagnosis of achalasia” according to Chicago Classification 4.0 does not contemplate the cases usually considered as “undetermined” phase. The first situation for inconclusive diagnosis is the presence of aperistalsis and IRP values at the upper limit of normal. Even though there was no definition for the upper limit, it is not uncommon to find with proved CDE and normal IRP values, especially in the setting of a hypotonic LES^9^. Moreover, surgeons are used to treat patients after the failure of endoscopic therapy when the parameter of the LES is lost^3^. Another situation is the presence of peristalsis when the manometry is repeated in a different position (supine vs. upright). In our opinion, this may represent a misinterpretation of the test rather than a peculiar diagnosis. Finally, there is reference to specific situations facing type III achalasia pattern that is not found in CDE since there is an impaired tonic effect of cholinergic nerves on the smooth muscle of the esophagus^10^.

Brazilian surgeons always believed on a complete workup to manage CDE rather than simply on manometric diagnosis^4^. Chicago Classification 4.0 only corroborates this belief.
